# PathTracer: High-sensitivity detection of differential pathway activity in tumours

**DOI:** 10.1038/s41598-019-52529-3

**Published:** 2019-11-08

**Authors:** Ståle Nygård, Ole Christian Lingjærde, Carlos Caldas, Eivind Hovig, Anne-Lise Børresen-Dale, Åslaug Helland, Vilde D. Haakensen

**Affiliations:** 1 Centre for Bioinformatics, Department of Informatics, University of Oslo, Oslo, Norway; 2 Bioinformatics core facility, Institute for Cancer Research, Oslo University Hospital, Oslo, Norway; 3 Department of Cancer Genetics, Institute for Cancer Research, Oslo University Hospital, Oslo, Norway; 4 KG Jebsen Centre for B-cell malignancies, Institute for Clinical Medicine, University of Oslo, Oslo, Norway; 5 Cancer Research UK, Cambridge Research Institute, Li Ka Shing Centre, University of Cambridge, Cambridge, UK; 6 Department of Tumor Biology, Institute for Cancer Research, Oslo University Hospital, Oslo, Norway; 7 Department of Oncology, Oslo University Hospital, Oslo, Norway; 8 Institute of Clinical Medicine, University of Oslo, Oslo, Norway

**Keywords:** Breast cancer, Computational science

## Abstract

Gene expression profiling of tumours is an important source of information for cancer patient stratification. Detecting subtle alterations of gene expression remains a challenge, however. Here, we propose a novel tool for high-sensitivity detection of differential pathway activity in tumours. For a pathway defined by a collection of genes, the samples are projected onto a low-dimensional manifold in the subspace spanned by those genes. For each sample, a score is next found by calculating the distance between each projected sample and the projection of a subgroup of reference samples. Depending on the aim of the analysis and the available data, the reference samples may represent e.g. normal tissue or tumour samples with a particular genotype or phenotype. The proposed tool, PathTracer, is demonstrated on gene expression data from 1952 invasive breast cancer samples, 10 DCIS, 9 benign samples and 144 tumour adjacent normal breast tissue samples. PathTracer scores are shown to predict survival, clinical subtypes, cellular proliferation and genomic instability. Furthermore, predictions are shown to outperform those obtained with other comparable methods.

## Introduction

Cancer initiation and progression involves deregulation of multiple signalling and metabolic pathways^1^. However, within the set of tumours being subject to deregulation of a particular pathway, the specific genomic and transcriptomic alterations can vary substantially. As a consequence, it can be challenging to link cancer progression to aberrations of a particular gene. Estimating the activity of pathways rather than single genes seeks to alleviate this problem by accumulating evidence of deregulation across all genes in a pathway, increasing the sensitivity compared to single-gene analyses. The identification of altered pathways may also facilitate development of personalised cancer treatments by identifying the main driving mechanisms in each tumour^2,3^. Notably, in recent years, cancer combination therapies have emerged that target specific pathways in parallel or multiple targets in the same pathway^4,5^. Carcinogenic alterations may occur at different molecular levels affecting the same genes and pathways. Analysis at the pathway level may identify pathways that are affected by alterations at different levels and in different genes in the same pathways, eg: DNA mutations and aberrations in some genes, silencing by methylation or miRNA-regulation of others. Gene sets or pathways have been analysed in various ways^6^. Gene set enrichment and gene ontology analyses identify genes that are significantly over-represented (enriched) in a gene list. The list is defined by an arbitrary threshold that may exclude significant genes. Construction of *de novo* networks of mutated/altered genes and their neighbours discards previous knowledge about biological function, but may identify known genetic associations. Finally, network-based modelling uses prior knowledge about networks/pathways to identify alterations in cancer.

*PARADIGM*^7^ was one of the first methods trying to meet the challenge of personalised pathway analysis, by describing the deregulation in a particular individual, as opposed to characterising a pathway’s activity for an entire sample set. Another such pathway-based method was *Pathifier*^2^. Pathifier calculates for every pathway a so-called Pathway Deregulation Score (PDS) representing the extent to which the pathway is deregulated in every individual sample. Pathifier utilises a non-linear analogy to principal components called principal curve^8^. Specifically, each tumour sample is projected onto the principal curve before calculating the distance to the centroid of the normal samples. A potential challenge using principal curves to represent the underlying data is that not all data can be represented by a single non-linear principal component. In that case, the behaviour of the principal curve approach will be unpredictable. Samples with high deregulation of a particular pathway will be located far from the normal samples in the multidimensional expression space, and we expect them to be located at the extreme end of the curve. However, if the principal curve does not represent the data well, this may not be the case (see file loops.html at github.com/staaln/pathtracer for several real-world examples). In such cases, the most deregulated samples may be located far from the extreme ends of the curve and then the distance from the tumour sample along the curve to the normal samples does not reflect the degree of deregulation. We propose to avoid this problem by using instead the Euclidean distance between two points on the principal curve, one representing the centroid of the normal samples and the other the projection of the tumour sample.

## Methods

The data input for the PathTracer method is a genome-wide gene expression profile (e.g from RNA-sequencing or microarray data) for a collection of samples of interest (e.g tumour samples), as well as for a collection of reference samples (e.g normal samples). First, expression values are gene-centered by subtraction of the mean and gene-scaled by division with the standard deviation. This standardisation is performed to ensure that all genes are on the same scale, otherwise the highly expressed genes will dominate in the principal components that will be constructed in the subsequent analysis. We assume the data to be normalised between samples prior to the analysis.

The method considers one pathway at a time, extracting gene expression data in all *n* samples for the genes in the pathway. For a pathway consisting of *p* genes, we thus obtain an *n* × *p* matrix *E* of gene expression values, and for the *i* th row (sample) we have a label *L*_*i*_ indicating whether the row represents a sample of interest or a reference sample. We here assume, without loss of generality, that the samples of interest are tumour samples and reference samples are from normal tissue (i.e *L*_*i*_ = “tumour” or *L*_*i*_ = “normal”, *i* = 1, …, *n*). We will only consider pathways above a given size, as pathways with very few genes are likely to give unstable results. We first derive a low-dimensional feature vector *z*_*i*_ ∈ *R*^*m*^ for the *i* th sample, where *m* ≪ *p*. The components of the vector *z*_*i*_ are the first *m* scores from a principal component analysis (PCA) derived from the gene expression matrix *E*. The number of components *m* may be fixed in advance or estimated from the data. We now have a reduced data matrix $\tilde{E}$ of dimension *n* × *m* where rows represent samples and columns represent features. The next step reduces the dimension even further from the *m*- dimensional feature vectors *z*_*i*_ to one-dimensional scores. To do this, a principal curve^8^ is fitted to the sample feature vectors *z*_*i*_. The first component in classical PCA is the line minimising the sum of squared distances from the data points to their orthogonal projections onto the line. A principal curve is analogous, except that the line is replaced by a smooth curve. Fitting a principal curve involves a trade-off between smoothness (degree of deviation from a straight line) and goodness-of-fit to the data. This trade-off is governed by a model parameter *λ* > 0 which is determined by cross-validation.

**Figure 1:**
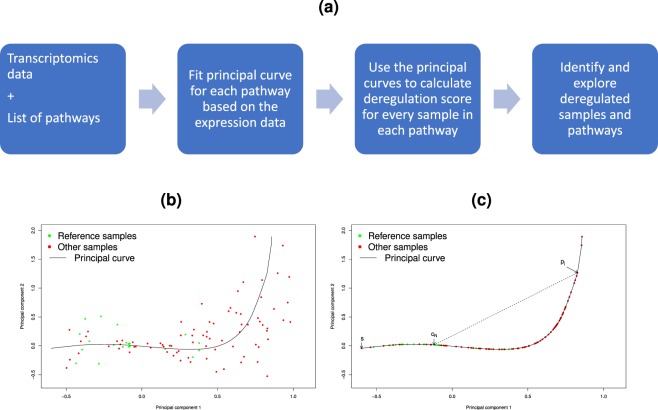
(**a**) Schematic overview of the PathTracer method. **(b)** Principal curve based on first two principal components. **(c)** Samples are projected onto the principal curve. Euclidean distance between the sample point *p*_*j*_ and the reference point *c*_*N*_, i.e the length of the dotted line, defines the deregulation score for sample *j*.

An overview of the method is illustrated in Fig. (1a). From the principal curve, we obtain a score for each sample as follows. Each *m*-dimensional feature vector *z*_*i*_ has an orthogonal projection *P*(*z*_*i*_) = *p*_*i*_ onto the principal curve (see Fig. 1b). If multiple projections exist, the nearest is selected. Let *O* denote an arbitrary point on the principal curve, and for each sample let *d*_*i*_ be the signed distance along the curve from the projection *p*_*i*_ to *O*. *O* can for example be taken as the starting point *s* of the principal curve. (Note that the *d*_*i*_'s then correspond to the pathway deregulation scores, PDS, in the Pathifier algorithm^2^). Let *k* be the index of the normal sample for which *d*_*k*_ = *median*(*d*_*i*_) where the median is calculated across all normal samples (if the number of normal samples is even, choose *k* to minimize the distance between the left and right hand side). Let *c*_*N*_ = *P*(*z*_*k*_) = *p*_*k*_ be the projection onto the principal curve of this normal sample. For the *i* th sample, define the score *e*_*i*_ = ||*p*_*i*_ − *c*_*N*_||, i.e the Euclidean distance between *p*_*i*_ and *c*_*N*_ (see Fig. 1c). Then scale the score so it becomes between 0 and 1 by dividing it by the maximum score value *e*_*max*_ = *max*_*k*∈1_, …, _*n*_*e*_*k*_. The PathTracer algorithm is summarised in the table below.

**Algorithm 1:**
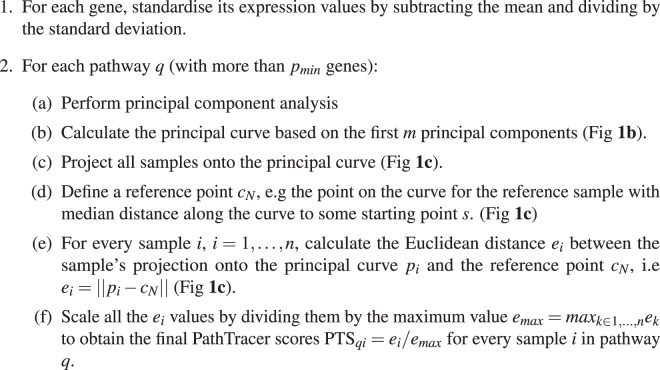
The PathTracer algorithm.

The number of principal components *m* in 2(b) must also be chosen. The optimal *m* will handle the bias-variance trade-off by including enough PCs to avoid underfitting, but at the same time avoiding overfitting by including enough PCs. We evaluated the performance in terms of survival prediction on training and test sets, and found that *m* = 4 gave the best results on the test set, but that results were quite stable in the range of *m* ∈ {3, …, 10}. See Supplementary Fig. 1. The minimum number of genes in a pathway *p*_*min*_ must also be chosen. In our application we have used *p*_*min*_ = 10, i.e about twice the number of principal components.

### Material

The breast cancer dataset used for the analyses has previously published as the Metabric dataset and the samples were collected in five different hospitals in the UK and Canada as previously described^9^. The METABRIC study collected samples from existing biobanks and the study was approved by the ethics committees in Cambridge and Vancouver ensuring that the methods were carried out in accordance with relevant guidelines and regulations. The expression data are available at the European Genome-phenome Archive (http://ebi.ac.uk/ega/) at the European Bioinformatics Institute (accession number EGAS00000000083). The Illumina HT-12 v3 platform was used for whole genome expression profiling^9^. This study has only used data available in the original publication. The cohort consists of a total of 2115 samples including 1952 invasive breast cancer samples, 10 DCIS, 9 benign samples and 144 tumour adjacent normal breast tissue samples.

## Results

### Breast cancer: normal vs tumour

The PathTracer algorithm was applied to whole genome gene expression data from the Metabric breast cancer cohort including 1952 breast cancer samples 10 DCIS, 9 benign samples and 144 adjacent normal breast tissue samples^9^. Pathways were downloaded from the Reactome database, extracting all Homo sapiens pathways (n = 1288) using the reactome.db R/Bioconductor package^10,11^.

The file loops.html (github.com/staaln/pathtracer/) shows a selected set of pathways where the original points are plotted together with the principal curve in rotatable 3d figures. For many of the pathways we see that the principal curve is quite far from many of the original points. We also see, as for example for the Defensin pathway, that the principal curve “back-flips”, and almost forms a loop formation. We note that even though the normal samples are quite close in terms of Euclidean distance, they are very from each other in terms of distance along the principal curve, and would thus get a very high PDS value. We also note that for this pathway the correlation between the PDS and the PTS is very low (Pearson correlation = −0.08). A low correlation between PDS and PTS will in general be indicative of such loop-like principal curve formation. We therefore calculated the Pearson correlation between PDS and PTS for all pathways (See Supplementary Fig. 2), and found that 72 pathways had correlation less than 0.5 and 459 less than 0.75, which indicates that quite a few pathways have a back-flipping principal curve, although for the majority of pathways this will not be a substantial problem.

**Figure 2:**
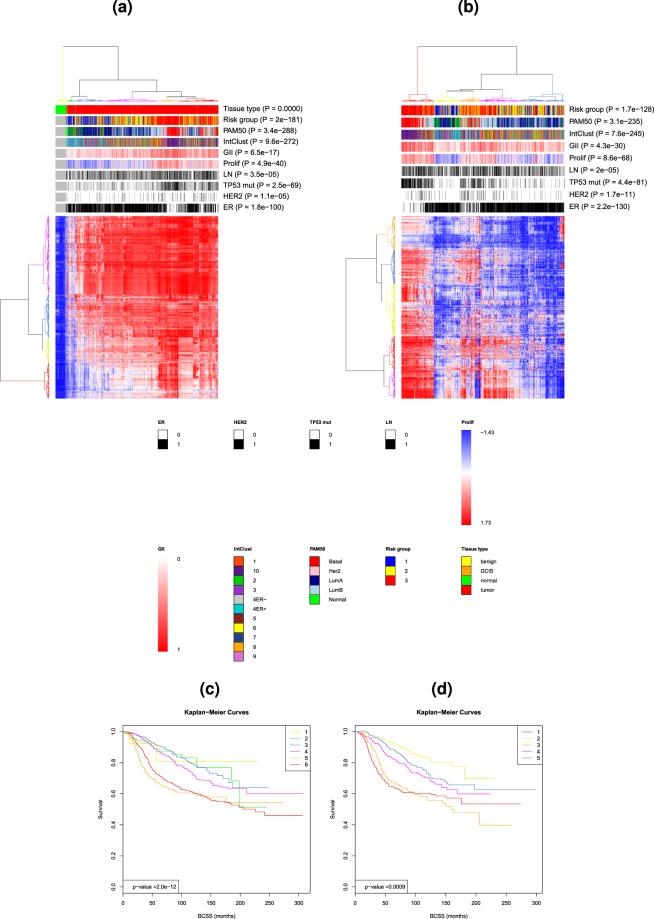
**(a)** Heatmap of pathways with AUC > 0.98 in separating normal (n = 144) and tumour samples (n = 1971). Blue and red colours indicate low and high PathTracer score, respectively. **(b)** Heatmap of pathways with AUC > 0.7 in separating tumour samples with (n = 387) and without (n = 1008) *TP53*-mutation. Blue and red colours indicate low and high PathTracer score, respectively. **(c)** Kaplan Meier curves of breast cancer specific surivival for the Metabric dataset for the six clusters identified in **(a)**. **(d)** Kaplan Meier curves of breast cancer specific survival for the Metabric dataset with tumour samples only, for the five clusters identified in **(b)**.

The samples were clustered to illustrate potential use of the PathTracer method. For illustrational purposes, we reduced the number of pathways substantially to include only pathways with PathTracer Scores (PTS) separating significantly between tumour and normal samples. In order to identify these pathways, we calculated the Area Under the Receiver Operating Characteristic (ROC) Curve (AUC). In a ROC curve, true positive rate or sensitivity (y-axis) is plotted against false positive rate or 1-specificity (x-axis) for the whole range of specificities. Here, tumour and normal samples were regarded as positives and negatives, respectively, and true positive rate and false positive rate for a given PTS threshold value was defined as the number of tumour samples and normal samples, respectively, with PTS over the threshold value. Based on the PTS of the pathways with AUC ≥ 0.98 (n = 322), we identified a total of six sample clusters highly associated with tissue type (*P* = 0.000), ER-status (*P* = 2.2*E* − 100), the expression based PAM50 breast cancer subtypes (*P* = 3.4*E* − 288), and the previously published Integrated Clusters subtypes^12^ (*P* = 9.6*E* − 272)(chi squared tests) (Fig. 2a). The six clusters were also associated with *TP53* mutation status, lymph node status, Risk of recurrence (ROR) group^13^, genomic instability index (GII), proliferation score and HER2-status (Fig. 2a). Breast cancer specific survival (BCSS) is significantly different between clusters (BCSS: *P* < 2.0*E* − 12; log-rank test) (Fig. 2c).

The heatmap in Fig. 2a reveals that most pathways are separating normal and tumour samples to a high degree. The first sample cluster consists mainly of normal samples (n = 140), but also contains 4 benign samples and 14 malignant tumour samples. These tumour samples are classified as normal-like (14/14) by PAM50 and as IC4 (14/14) by IntClust, of which 12 are IC4ER+ and 2 IC4ER−. The samples classified as poor risk according to ‘Risk of Recurrence’ (ROR) cluster with the poor prognosis subtypes basal-like, HER2-enriched and IC5 in cluster 5 and 6 (orange and red). The orange samples (cluster 5) consist mainly of samples classified as basal-like (PAM50 red) and IC5 (IntClust purple), both characterised by poor prognosis (ref Curtis and Sorlie PNAS 2001). This sample cluster sees higher PTS than the other sample clusters in pathways involved in cell cycle (APC/C-mediated degradation and mitosis), transcription (polymerase), apoptosis and DNA-repair (translesion) (Fig. 2a). The green and blue sample clusters 2 and 3 are characterised by low risk of relapse, low proliferation, genomic aberration and *TP53* mutation. For sample cluster 2 (green) most samples belong to IC4ER+ and IC3 and normal-like or luminal A subtypes, while sample cluster 3 (blue) contains mainly samples of IC3 and luminal A subtypes. Both the gene-expression based PAM50 subtypes and the IntClust (based both on gene expression and genomic data) are highly associated with the clustering observed based on pathway deregulation (p = 3.4 × 10–288 and p = 9.6 × 10–272, Fig. 2a). Figure (2c) shows Kaplan-Meier plots with p-value from log-rank test for survival-differences between the clusters in Fig. (2a)).

**Figure 3:**
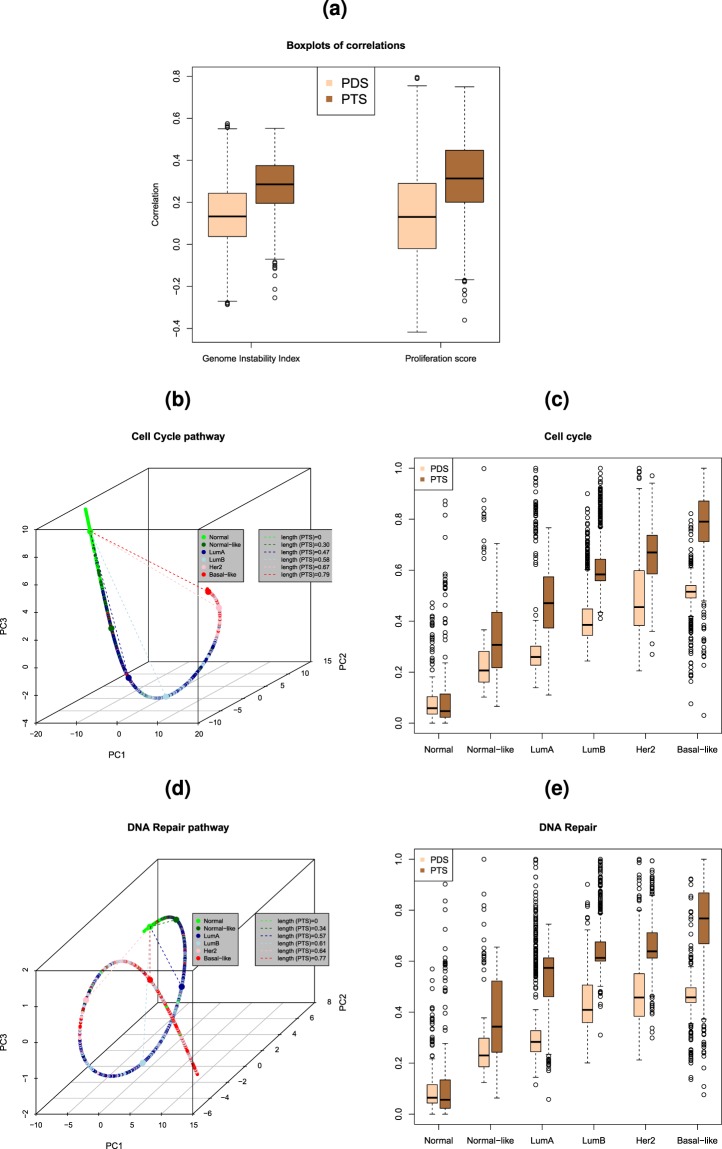
**(a)** Box plots of the Pearson correlation between deregulation scores (PTS/PDS) and Genome Instability Index (GII) (left) and proliferation index (right) using the Metabric dataset with n = 1971 tumour samples and n = 144 normal samples and 1288 human Reactome pathways. **(b)** Projection of samples onto the principal curve for the Cell cycle pathway using the Metabric dataset. The samples are coloured according to PAM50 subtype. The median PahtTracer score for each subtype is given by the length of the dotted lines. **(c)** Comparison of Pathifier (PDS) and PahtTracer (PTS) deregulation scores for each of the PAM50 subtypes using the Metabric dataset. **(d)** Projection of samples onto the principal curve for the DNA repair pathway using the Metabric dataset. The samples are coloured according to PAM50 subtype. The median PathTracer score for each subtype is given by the length of the dotted lines. **(e)** Comparison of Pathifier (PDS) and PahtTracer (PTS) deregulation scores for each of the PAM50 subtypes using the Metabric dataset.

PathTracer deregulation scores were correlated with proliferation score, genome instability index (GII) (Fig. 3a) and overall survival (Table 1). To determine GII, we first applied the Allele Specific Copy number Aberrations in Tumours (ASCAT) algorithm^14^ to Affymetrix SNP6 data from all the samples to estimate the exact copy number along the genome. We next defined GII as the proportion of the genome with estimated copy number deviating from the average copy number in either direction by at least 0.5. The proliferation score was calculated as the mean expression of the 11 proliferation genes *CCNB1*, *UBE2C*, *BIRC5*, *KNTC2*, *CDC20*, *PTTG1*, *RRM2*, *MKI67*, *TYMS*, *CEP55*, and *CDCA1*, as previously described^13^. Gene centering was performed before calculation of the proliferation score. The Pearson correlation between PathTracer scores (PTS) and Genome Instability Index (GII) across pathways (median: 0.29, IQR: 0.20–0.37) was significantly higher than the correlation between the Pathifier deregulation scores PDS (PDS) and GII (median:0.13, IQR:0.04–0.25) (*P* < 2.2·10^−16^; Fig. 3a). The Pearson correlation between PTS and the proliferation score (median: 0.31, IQR: 0.20–0.45) was also significantly higher than the correlation between PDS and proliferation score (median: 0.13, IQR: −0.02–0.29) (*P* < 2.2·10^−16^; Fig. 3a. We next performed Cox regression on breast cancer specific survival calculated from time of diagnosis, using either PTS or PDS as the covariate. The proportion of pathways having a significant association to survival (*P* < 0.05) is higher for PTS than for PDS for a range of different significance thresholds (see Table 1). The deregulation scores were also correlated to PAM50 subtypes^13^ using two well-known pathways, namely Cell Cycle pathway and DNA Repair pathway. Figure 3b,d show the samples projected onto the principal curves and coloured according to PAM50 subtypes. After the normal samples, which are located mostly at the start of the curves, we find predominantly normal-like samples followed by LumA, LumB, Her2, and finally, Basal-like samples. This is an ordering which fits well with severity of disease. The boxplots in Fig. 3c,e include also the PDS values for comparison. We see that the PTS values in general are higher than the PDS values, but more notably for the LumA, LumB, Basal-like and Her2 subtypes, implying that the PTS values are more highly correlated with cancer severity (according to PAM5 subtype) than the PDSs.

**Table 1:** Proportion of significant pathways in Cox regression of overall survival.

Threshold	PDS	PTS	Ratio
0.05	0.537	0.680	1.186
0.04	0.514	0.650	1.265
0.03	0.481	0.615	1.279
0.02	0.443	0.559	1.262
0.01	0.375	0.469	1.251
0.005	0.313	0.386	1.233
0.001	0.198	0.237	1.196
0.0001	0.078	0.119	1.525
0.00001	0.013	0.051	3.920

The first column in the table is the threshold used to determine if a Cox-regression P-value is significant, the second column is the proportion of pathways significant with Pathway Deregulation Score (PDS) as covariate, the third column is the proportion of pathways significant with PathTracer Score (PTS) as covariate, and the fourth column is the ratio between the third and the second column. In the figure the threshold values (first column) are plotted against the proportion of significant pathways PDS (black colour) and PTS (red colour).

### Breast cancer: *TP53* mutated vs. *TP53* wild-type

To illustrate an alternative use of the method (without reference to normal tissue samples), we have identified PTSs comparing *TP53* mutated and wild-type samples for tumours in the Metabric dataset using wild-type samples as reference group. Figure 2b illustrates clustering of the samples based on pathways with an AUC > 0.7 (n = 364). The red sample cluster 1 is characterised by high PTS, ROR-risk, GII and proliferation, a large proportion of TP53-mutation and an enrichment in basal-like and IC10 subtypes. Kaplan-Meier plots confirm the poor prognosis (Fig. 2d). The samples in cluster 1 are characterised by high PTS in pathways related to cell cycle, gene expression and metabolism of proteins. The pathways constituting the lower 8 lines of the heatmap show a high PTS in both poor prognosis sample clusters (red sample cluster 1 and the pink sample cluster 4) and are all involved in regulation of cell cycle, specifically p53-independent DNA damage response, APC/C-mediated degradation and CDK-mediated removal of CDC6). Two sample clusters are characterised by a majority of *TP53* wild-type ER positive samples; cluster 2 (yellow) and cluster 5 (blue). Both are dominated by luminal A and IC3 samples and have a good prognosis (Fig. 2d). The yellow sample cluster 2 has a higher representation of normal-like and IC4+ samples and the blue sample cluster 5 has a higher representation of luminal B, IC7 and IC8 samples. The pathways in the lower pink pathway cluster 5 have a high PTS in the poor prognosis red sample cluster 1 and includes mainly pathways involved in cell cycle. These pathways also have a high PTS in those samples belonging to the pink sample cluster 4 that have a high risk of relapse according to ROR.

## Discussion

Analysis of pathway deregulation in cancer captures alterations at different molecular levels as well as in different genes in the pathway and may give an overview of biological alterations in carcinogenesis and characteristics particular to a specific tumour. Identifying pathway deregulation allows identification of combined alterations that affect the cancer biology more than identifying single mutations. This is important for a broader understanding of the biology, but also for the development of new treatment strategies and for understanding treatment failure.

PathTracer identifies deregulation of a user-defined list of input pathways in cancer samples compared with a specified set of normal (reference) samples. Use of the method may be exploratory by including a wide range of pathways to identify which are most deregulated. The method may also be used for studying the behaviour of a specific set of pathways related to one or a few biological functions.

The motivation for developing PathTracer was the worry that previous methods would estimate an unjustified high deregulation score to certain pathways. The presented method is similar to the Pathifier method^2^, in that it utilizes principal curves to obtain deregulation scores, but differ in many ways, affecting both robustness of results, computational time, and output visualisation. Most importantly, we have implemented a novel distance measure method to address the problem of pathways where the principal curve goes in a loop. Pathifier will in such situations estimate the deregulation score along the curve and assign a high deregulation score to a sample located at the tail of the curve but close to the reference samples. The biological meaning of such a score is questionable. By using the Euclidean distance from the projection of the sample onto the principal curve to the reference point *c*_*N*_, the samples will get a deregulation score that reflects the distance from the sample to the reference samples in a multidimensional space and which is more likely to reflect the underlying biology. In addition to the problem with principal curves going in loops, one may get unreliable results if the curves are very ragged (non-smooth), because such curves will generate long distances along the curves (and very high deregulation score), even though the Euclidean distances to the reference point may not be long. To further improve robustness, PathTracer has an optional pre-filtering step where pathways with fewer than *k* genes are omitted from the analysis (in the reported results, we use *k* = 10). The rationale behind this pre-filtering is that pathways consisting of very few genes are less likely to benefit from the dimension reduction step in PathTracer. There is a trade-off involved here, however, as one may risk missing key pathways by such filtering. We would therefore recommend that use of pre-filtering is used with caution if one suspects that important pathways may thus be discarded. Further, as pathway analyses tend to be extremely time consuming, substantial changes to decrease computational time has been implemented. The most time-consuming part in the Pathifier method is a cross-validation procedure to find the optimal number of principal components (PCs). By instead locking the number of PCs, we reduce the computational time about 10-fold. In addition, we have added the possibility of performing parallelization, which enables the user to run the analysis on several cores simultaneously, drastically reducing the calculation time. Several features enabling data presentation have been incorporated in the PathTracer method, including generation of a heatmap with clustering of samples and deregulation scores including significance testing between the input categories for each pathway. A principal curve plot for each pathway may help interpret the results and help the researcher understand the biology underlying the figures produced by the algorithm.

The PathTracer method was applied to a set of breast cancer samples to illustrate its use. Survival and the biological features genome instability index and proliferation score show increased correlation with PTS (PathTracer) compared with PDS (Pathifier). Clustering based on PTS identified clusters with varying degree of pathway deregulation and a high correlation with clinical and biological features.

## Supplementary information

Supplementary figure 1

Supplementary figure 2

## Data Availability

We have developed an R-package called PathTracer available on GitHub (github.com/staaln/pathtracer).
