# Supplementary material for: PathTracer: High-sensitivity detection of differential pathway activity in tumours
Source: Sci Rep. 2019 Nov 8;9:16332. doi: 10.1038/s41598-019-52529-3 (PMC6841931; doi:10.1038/s41598-019-52529-3)
Supplement: Supplementary file 1 — Supplementary figure 1 [file 41598_2019_52529_MOESM1_ESM.pdf]

PathTracer: High-sensitivity detection of differential pathway activity in tumours

Ståle Nygård[1,2,*], Ole Christian Lingjærde[1,3,4,*], Carlos Caldas[5], Eivind Hovig[1,6], Anne-Lise Børresen-Dale[3], ˚ Aslaug Helland[3,7,8], and Vilde D. Haakensen[3,7,†]

[1]Centre for Bioinformatics, Department of Informatics, University of Oslo, Oslo, Norway
[2]Bioinformatics core facility, Institute for Cancer Research, Oslo University Hospital, Oslo, Norway
[3]Department of Cancer Genetics, Institute for Cancer Research, Oslo University Hospital, Oslo, Norway
[4]KG Jebsen Centre for B-cell malignancies, Institute for Clinical Medicine, University of Oslo, Norway
[5]Cancer Research UK, Cambridge Research Institute, Li Ka Shing Centre, University of Cambridge, Cambridge, UK
[6]Department of Tumor Biology, Institute for Cancer Research, Oslo University Hospital, Oslo, Norway
[7]Department of Oncology, Oslo University Hospital, Oslo, Norway
[8]Institute of Clinical Medicine, University of Oslo, Oslo, Norway
[*]These authors contributed equally to this study
[†]Corresponding author vilde.haakensen@gmail.com

Comparison of Cox P-values on training and test data set for novel PathTracer score. Left panel: Percentage of significant pathways after Bonferroni correction on training data set (n = 1465 patients). Right panel: Percentage of significant pathways after Bonferroni correction on test data set (n = 650 patients).

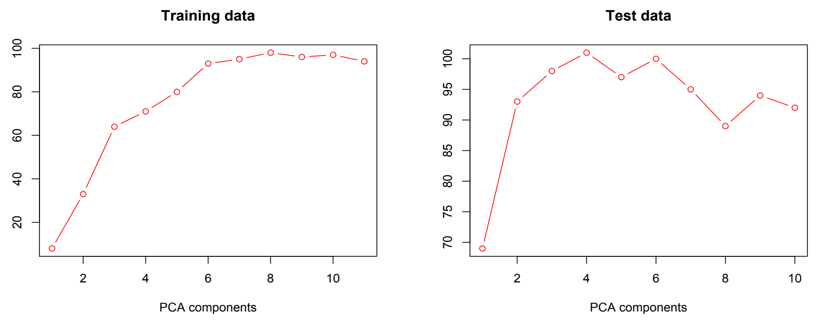

Observation: k = 4 PCA components seems to perform best on the test data set.

Evaluation of number of principal components performed by splitting the metabric breast cancer dataset into training and test samples. In the test data, samples were projected into the principal curve learned from the training data.
