# Supplementary material for: PathTracer: High-sensitivity detection of differential pathway activity in tumours
Source: Sci Rep. 2019 Nov 8;9:16332. doi: 10.1038/s41598-019-52529-3 (PMC6841931; doi:10.1038/s41598-019-52529-3)
Supplement: Supplementary file 2 — Supplementary figure 2 [file 41598_2019_52529_MOESM2_ESM.pdf]

PathTracer: High-sensitivity detection of differential pathway activity in tumours

Ståle Nygård[1,2,*], Ole Christian Lingjærde[1,3,4,*], Carlos Caldas[5], Eivind Hovig[1,6], Anne-Lise Børresen-Dale[3], ˚ Aslaug Helland[3,7,8], and Vilde D. Haakensen[3,7,†]

[1]Centre for Bioinformatics, Department of Informatics, University of Oslo, Oslo, Norway
[2]Bioinformatics core facility, Institute for Cancer Research, Oslo University Hospital, Oslo, Norway
[3]Department of Cancer Genetics, Institute for Cancer Research, Oslo University Hospital, Oslo, Norway
[4]KG Jebsen Centre for B-cell malignancies, Institute for Clinical Medicine, University of Oslo, Norway
[5]Cancer Research UK, Cambridge Research Institute, Li Ka Shing Centre, University of Cambridge, Cambridge, UK
[6]Department of Tumor Biology, Institute for Cancer Research, Oslo University Hospital, Oslo, Norway
[7]Department of Oncology, Oslo University Hospital, Oslo, Norway
[8]Institute of Clinical Medicine, University of Oslo, Oslo, Norway
[*]These authors contributed equally to this study
[†]Corresponding author vilde.haakensen@gmail.com

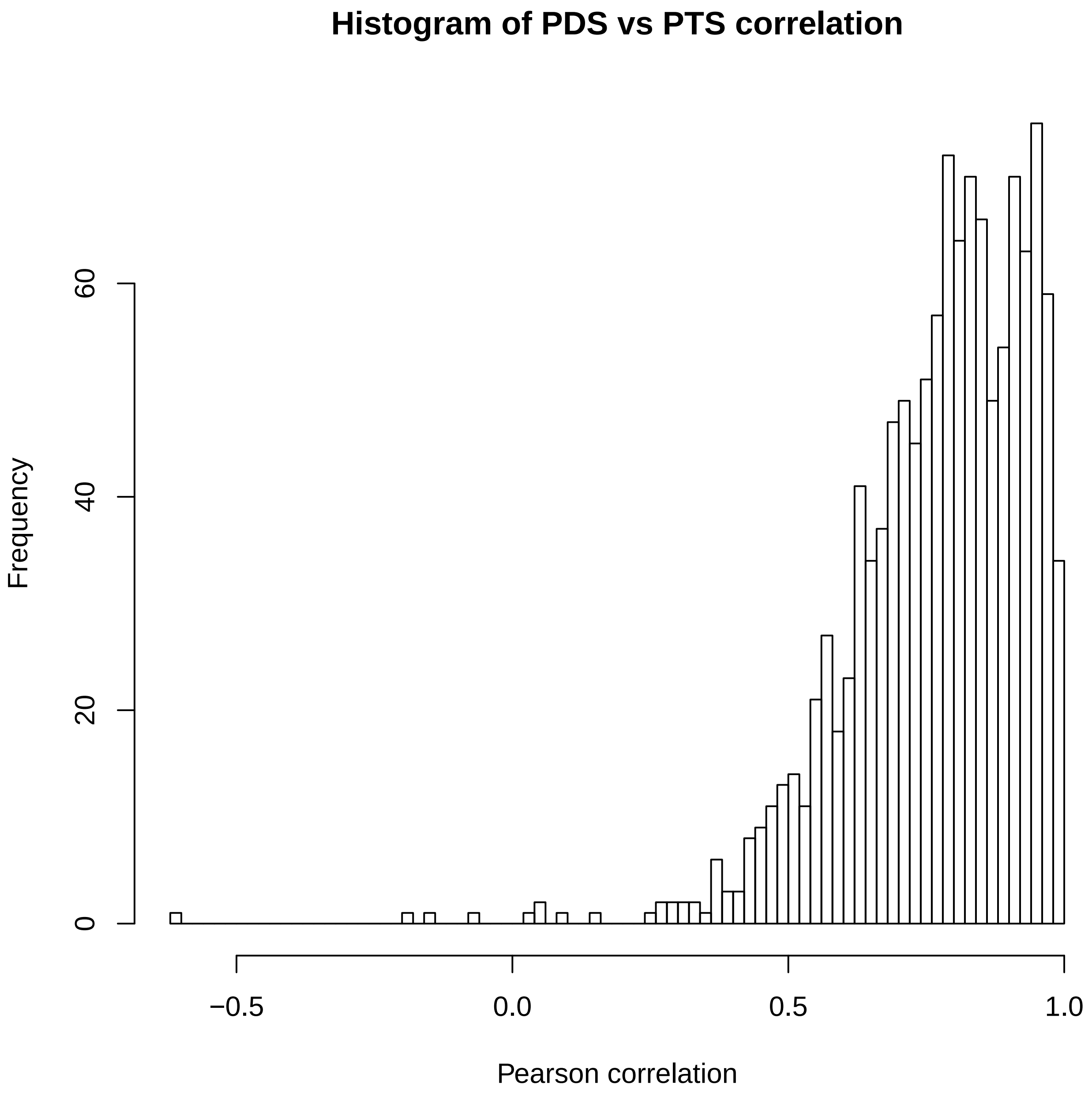

Histogram of correlation between pathifier deregulation scores (PDS) and PathTracer scores (PTS) based on the metabric breast cancer dataset.
